# Supplementary material for: Neighborhood environment and incident diabetes, a neighborhood environment-wide association study (‘NE-WAS’): Results from the Hispanic Community Health Study/Study of Latinos (HCHS/SOL)
Source: PLoS One. 2025 Jul 29;20(7):e0329282. doi: 10.1371/journal.pone.0329282 (PMC12306752; doi:10.1371/journal.pone.0329282)
Supplement: S1 Table — (DOCX) [file pone.0329282.s001.docx]

**S1 Table**. List of neighborhood measures used in analysis, if they were log transformed for analysis, their bin categorization, and their data source.

| Neighborhood Measure | Log Transformed | Bin | Data Source |
| --- | --- | --- | --- |
| Number of households in tract | Yes | Demographics and Households | NaNDA, Internet Access, American Community Survey (2015-2019) |
| % of households with cellular data plan |  | Demographics and Households | NaNDA, Internet Access, American Community Survey (2015-2019) |
| % of households with dsl/cable broadband |  | Demographics and Households | NaNDA, Internet Access, American Community Survey (2015-2019) |
| % of households with satellite internet |  | Demographics and Households | NaNDA, Internet Access, American Community Survey (2015-2019) |
| % of households with any computing device and any type of broadband |  | Demographics and Households | NaNDA, Internet Access, American Community Survey (2015-2019) |
| % of households with internet access without a subscription |  | Demographics and Households | NaNDA, Internet Access, American Community Survey (2015-2019) |
| % of households with laptop/desktop |  | Demographics and Households | NaNDA, Internet Access, American Community Survey (2015-2019) |
| % of households with no computing device |  | Demographics and Households | NaNDA, Internet Access, American Community Survey (2015-2019) |
| Population in 1km buffer | Yes | Demographics and Households | 2010 US Census |
| Tract level median year structure built |  | Demographics and Households | 2010 US Census |
| Tract level median value for specified owner-occupied housing units |  | Demographics and Households | 2010 US Census |
| Tract level % under 5 years |  | Demographics and Households | 2010 US Census |
| Tract level % 10 to 14 years |  | Demographics and Households | 2010 US Census |
| Tract level % 15 to 17 years |  | Demographics and Households | 2010 US Census |
| Tract level % 18 to 24 years | Yes | Demographics and Households | 2010 US Census |
| Tract level % 25 to 34 years | Yes | Demographics and Households | 2010 US Census |
| Tract level % 35 to 44 years |  | Demographics and Households | 2010 US Census |
| Tract level % 45 to 54 years |  | Demographics and Households | 2010 US Census |
| Tract level % 5 to 9 years |  | Demographics and Households | 2010 US Census |
| Tract level % 55 to 64 years |  | Demographics and Households | 2010 US Census |
| Tract level % 65 to 74 years | Yes | Demographics and Households | 2010 US Census |
| Tract level % 75 to 84 years | Yes | Demographics and Households | 2010 US Census |
| Tract level % 85 years and over |  | Demographics and Households | 2010 US Census |
| Tract level % foreign born |  | Demographics and Households | 2010 US Census |
| Tract level % Hispanic |  | Demographics and Households | 2010 US Census |
| Tract level % occupied housing units using electricity for heating fuel | Yes | Demographics and Households | 2010 US Census |
| Tract level % occupied housing units using gas for heating fuel |  | Demographics and Households | 2010 US Census |
| Tract level % occupied housing units using oil for heating fuel |  | Demographics and Households | 2010 US Census |
| Tract level % occupied housing units using coal, coke or wood for heating fuel |  | Demographics and Households | 2010 US Census |
| Tract level % occupied housing units occupied by renter |  | Demographics and Households | 2010 US Census |
| Tract level % of housing units with 1 unit | Yes | Demographics and Households | 2010 US Census |
| Tract level % of housing units with 10 to 49 units |  | Demographics and Households | 2010 US Census |
| Tract level % of housing units with 2 units |  | Demographics and Households | 2010 US Census |
| Tract level % of housing units with 3 to 9 units |  | Demographics and Households | 2010 US Census |
| Tract level % of housing units with 50 or more units |  | Demographics and Households | 2010 US Census |
| Tract level % of housing units that are mobile home |  | Demographics and Households | 2010 US Census |
| Tract level % of housing units that are boats, RV, vans, other |  | Demographics and Households | 2010 US Census |
| Tract level % population 15 years and over now married (not including separated) |  | Demographics and Households | 2010 US Census |
| Tract level % population 15 years and over divorced |  | Demographics and Households | 2010 US Census |
| Tract level % population 15 years and over never married |  | Demographics and Households | 2010 US Census |
| Tract level % population 15 years and over separated |  | Demographics and Households | 2010 US Census |
| Tract level % population 15 years and over widowed |  | Demographics and Households | 2010 US Census |
| Tract level percent Asian alone |  | Demographics and Households | 2010 US Census |
| Tract level percent Black or African-American alone | Yes | Demographics and Households | 2010 US Census |
| Tract level % two or more races | Yes | Demographics and Households | 2010 US Census |
| Tract level % American Indian and Alaska native alone |  | Demographics and Households | 2010 US Census |
| Tract level % other |  | Demographics and Households | 2010 US Census |
| Tract level % native Hawaiian and other Pacific Islander |  | Demographics and Households | 2010 US Census |
| Tract level % White alone |  | Demographics and Households | 2010 US Census |
| Tract level % Male |  | Demographics and Households | 2010 US Census |
| Tract level population 25 years and older with a bachelor's degree | Yes | Education, Employment, and Income | 2010 US Census |
| Tract level population 25 years and older high school graduate or equivalency |  | Education, Employment, and Income | 2010 US Census |
| Tract level population 25 years and older less than high school |  | Education, Employment, and Income | 2010 US Census |
| Tract level population 25 years and older with a doctorate degree |  | Education, Employment, and Income | 2010 US Census |
| Tract level population 25 years and older with a professional school degree |  | Education, Employment, and Income | 2010 US Census |
| Tract level population 25 years and older with some college |  | Education, Employment, and Income | 2010 US Census |
| Tract level population 16 to 19 years not high school graduate, not enrolled (dropped out) | Yes | Education, Employment, and Income | 2010 US Census |
| Tract level percent employed civilian population 16 years and over in construction | Yes | Education, Employment, and Income | 2010 US Census |
| Tract level percent employed civilian population 16 years and over in educational services, and health care and social assistance | Yes | Education, Employment, and Income | 2010 US Census |
| Tract level percent employed civilian population 16 years and over in agriculture, forestry, fishing and hunting, and mining |  | Education, Employment, and Income | 2010 US Census |
| Tract level percent employed civilian population 16 years and over in finance and insurance, and real estate and rental and leasing | Yes | Education, Employment, and Income | 2010 US Census |
| Tract level percent employed civilian population 16 years and over in arts, entertainment, and recreation, and accommodation and food services |  | Education, Employment, and Income | 2010 US Census |
| Tract level percent employed civilian population 16 years and over in information |  | Education, Employment, and Income | 2010 US Census |
| Tract level percent employed civilian population 16 years and over in manufacturing |  | Education, Employment, and Income | 2010 US Census |
| Tract level percent employed civilian population 16 years and over in other services except public administration | Yes | Education, Employment, and Income | 2010 US Census |
| Tract level percent employed civilian population 16 years and over in professional, scientific, and management, and administrative, and waste management services | Yes | Education, Employment, and Income | 2010 US Census |
| Tract level percent employed civilian population 16 years and over in public administration | Yes | Education, Employment, and Income | 2010 US Census |
| Tract level percent employed civilian population 16 years and over in retail trade |  | Education, Employment, and Income | 2010 US Census |
| Tract level percent employed civilian population 16 years and over in wholesale trade | Yes | Education, Employment, and Income | 2010 US Census |
| Tract level percent employed civilian population 16 years and over in transportation and warehousing, and utilities |  | Education, Employment, and Income | 2010 US Census |
| Tract level % of households with income less than $100,000 |  | Education, Employment, and Income | 2010 US Census |
| Tract level % of households with income less than $150,000 |  | Education, Employment, and Income | 2010 US Census |
| Tract level % of households with income less than $200,000 |  | Education, Employment, and Income | 2010 US Census |
| Tract level % of households with income less than $25,000 |  | Education, Employment, and Income | 2010 US Census |
| Tract level % population 16 years and over in labor force |  | Education, Employment, and Income | 2010 US Census |
| Tract level population for whom poverty status is determined under 1.00 |  | Education, Employment, and Income | 2010 US Census |
| Tract level population for whom poverty status is determined 1.00 to 1.99 |  | Education, Employment, and Income | 2010 US Census |
| Tract level population for whom poverty status is determined 2.00 and over |  | Education, Employment, and Income | 2010 US Census |
| Tract level % civilian population 16 years and over in labor force: unemployed |  | Education, Employment, and Income | 2010 US Census |
| Tract level % civilian male population 16 years and over in labor force: unemployed |  | Education, Employment, and Income | 2010 US Census |
| Number of supermarkets/grocery stores per sq. mile with 2+ employees | Yes | Goods and Services | NaNDA, Grocery Stores by Census Tract, US Census 2010 and National Establishment Time Series, 2010 |
| Number of specialty food stores per sq. mile with 2+ employees | Yes | Goods and Services | NaNDA, Grocery Stores by Census Tract, US Census 2010 and National Establishment Time Series, 2010 |
| Number of warehouse clubs/supercenters per sq. mile with 2+ employees |  | Goods and Services | NaNDA, Grocery Stores by Census Tract, US Census 2010 and National Establishment Time Series, 2010 |
| Number of post offices/postal service facilities per sq. mile with 2+ employees |  | Goods and Services | NaNDA, Post Offices and Banks by Census Tract, North American Industry Classification System and National Establishment Time Series, 2010 |
| Number of commercial banks per sq. mile with 2+ employees | Yes | Goods and Services | NaNDA, Post Offices and Banks by Census Tract, North American Industry Classification System and National Establishment Time Series, 2010 |
| Number of savings and loan banks per sq. mile with 2+ employees |  | Goods and Services | NaNDA, Post Offices and Banks by Census Tract, North American Industry Classification System and National Establishment Time Series, 2010 |
| Number of credit unions per sq. mile with 2+ employees |  | Goods and Services | NaNDA, Post Offices and Banks by Census Tract, North American Industry Classification System and National Establishment Time Series, 2010 |
| Number of bars per sq. mile with 2+ employees | Yes | Goods and Services | NaNDA, Eating and Drinking Places by Census Tract, North American Industry Classification System and National Establishment Time Series, 2010 |
| Number of full-service restaurants per sq. mile with 2+ employees | Yes | Goods and Services | NaNDA, Eating and Drinking Places by Census Tract, North American Industry Classification System and National Establishment Time Series, 2010 |
| Number of fast food restaurants per sq. mile with 2+ employees | Yes | Goods and Services | NaNDA, Eating and Drinking Places by Census Tract, North American Industry Classification System and National Establishment Time Series, 2010 |
| Number of coffee shops per sq. mile with 2+ employees |  | Goods and Services | NaNDA, Eating and Drinking Places by Census Tract, North American Industry Classification System and National Establishment Time Series, 2010 |
| Density of furniture/home furnishing stores in 2010 (count/total pop) |  | Goods and Services | NaNDA, Retail Establishments by Census Tract, North American Industry Classification System and National Establishment Time Series, 2010 |
| Density of electronics and appliance stores in 2010 (count/total pop) |  | Goods and Services | NaNDA, Retail Establishments by Census Tract, North American Industry Classification System and National Establishment Time Series, 2010 |
| Density of building and garden supply stores in 2010 (count/total pop) |  | Goods and Services | NaNDA, Retail Establishments by Census Tract, North American Industry Classification System and National Establishment Time Series, 2010 |
| Density of liquor stores in 2010 (count/total pop) |  | Goods and Services | NaNDA, Retail Establishments by Census Tract, North American Industry Classification System and National Establishment Time Series, 2010 |
| Density of clothing and accessories stores in 2010 (count/total pop) |  | Goods and Services | NaNDA, Retail Establishments by Census Tract, North American Industry Classification System and National Establishment Time Series, 2010 |
| Density of sporting goods, hobby, musical instrument, and book stores in 2010 (count/total pop) |  | Goods and Services | NaNDA, Retail Establishments by Census Tract, North American Industry Classification System and National Establishment Time Series, 2010 |
| Density of general stores in 2010 (count/total pop) |  | Goods and Services | NaNDA, Retail Establishments by Census Tract, North American Industry Classification System and National Establishment Time Series, 2010 |
| Density of used merchandise stores in 2010 (count/total pop) |  | Goods and Services | NaNDA, Retail Establishments by Census Tract, North American Industry Classification System and National Establishment Time Series, 2010 |
| Density of tobacco stores in 2010 (count/total pop) |  | Goods and Services | NaNDA, Retail Establishments by Census Tract, North American Industry Classification System and National Establishment Time Series, 2010 |
| Density of libraries and archives in 2010 (counts/pop) |  | Goods and Services | NaNDA, Arts, Entertainment, and Recreation Organizations by Census Tract, North American Industry Classification System and National Establishment Time Series, 2010 |
| Density of social services organizations in 2010 (count/total pop) |  | Goods and Services | NaNDA, Social Services Organizations by Census Tract, North American Industry Classification System and National Establishment Time Series, 2010 |
| Density of child/youth services in 2010 (count/total pop) |  | Goods and Services | NaNDA, Social Services Organizations by Census Tract, North American Industry Classification System and National Establishment Time Series, 2010 |
| Density of elderly/disability services in 2010 (count/total pop) |  | Goods and Services | NaNDA, Social Services Organizations by Census Tract, North American Industry Classification System and National Establishment Time Series, 2010 |
| Density of other individual/family services in 2010 (count/total pop) |  | Goods and Services | NaNDA, Social Services Organizations by Census Tract, North American Industry Classification System and National Establishment Time Series, 2010 |
| Density of community food services in 2010 (count/total pop) |  | Goods and Services | NaNDA, Social Services Organizations by Census Tract, North American Industry Classification System and National Establishment Time Series, 2010 |
| Density of emergency relief services in 2010 (count/total pop) |  | Goods and Services | NaNDA, Social Services Organizations by Census Tract, North American Industry Classification System and National Establishment Time Series, 2010 |
| Density of job training services in 2010 (count/total pop) |  | Goods and Services | NaNDA, Social Services Organizations by Census Tract, North American Industry Classification System and National Establishment Time Series, 2010 |
| Density of child day care services in 2010 (count/total pop) |  | Goods and Services | NaNDA, Social Services Organizations by Census Tract, North American Industry Classification System and National Establishment Time Series, 2010 |
| Density of personal care establishments in 2010 |  | Goods and Services | NaNDA, Personal Services by Census Tract, North American Industry Classification System and National Establishment Time Series, 2010 |
| Density of laundries and dry cleaners in 2010 |  | Goods and Services | NaNDA, Personal Services by Census Tract, North American Industry Classification System and National Establishment Time Series, 2010 |
| Density of religious organization in 2010 (count/total pop) |  | Goods and Services | NaNDA, Religious, Civic, and Social Organizations by Census Tract, North American Industry Classification System and National Establishment Time Series, 2010 |
| Density of civic and social organization in 2010 (count/total pop) |  | Goods and Services | NaNDA, Religious, Civic, and Social Organizations by Census Tract, North American Industry Classification System and National Establishment Time Series, 2010 |
| Total justice/public safety organizations in the tract in 2010 |  | Goods and Services | NaNDA, Justice and Public Safety Organizations by Census Tract, North American Industry Classification System and National Establishment Time Series, 2010 |
| Number of supermarkets/grocery stores per 1000 people with 2+ employees | Yes | Goods and Services | NaNDA, Grocery Stores by Census Tract, US Census 2010 and National Establishment Time Series, 2010 |
| Number of specialty food stores per 1000 people with 2+ employees | Yes | Goods and Services | NaNDA, Grocery Stores by Census Tract, US Census 2010 and National Establishment Time Series, 2010 |
| Number of post offices/postal service facilities per 1000 people with 2+ employees |  | Goods and Services | NaNDA, Post Offices and Banks by Census Tract, North American Industry Classification System and National Establishment Time Series, 2010 |
| Number of commercial banks per 1000 people with 2+ employees |  | Goods and Services | NaNDA, Post Offices and Banks by Census Tract, North American Industry Classification System and National Establishment Time Series, 2010 |
| Number of savings and loan banks per 1000 people with 2+ employees |  | Goods and Services | NaNDA, Post Offices and Banks by Census Tract, North American Industry Classification System and National Establishment Time Series, 2010 |
| Number of credit unions per 1000 people with 2+ employees |  | Goods and Services | NaNDA, Post Offices and Banks by Census Tract, North American Industry Classification System and National Establishment Time Series, 2010 |
| Number of bars per 1000 people with 2+ employees |  | Goods and Services | NaNDA, Eating and Drinking Places by Census Tract, North American Industry Classification System and National Establishment Time Series, 2010 |
| Number of full-service restaurants per 1000 people with 2+ employees | Yes | Goods and Services | NaNDA, Eating and Drinking Places by Census Tract, North American Industry Classification System and National Establishment Time Series, 2010 |
| Number of fast food restaurants per 1000 people with 2+ employees | Yes | Goods and Services | NaNDA, Eating and Drinking Places by Census Tract, North American Industry Classification System and National Establishment Time Series, 2010 |
| Number of coffee shops per 1000 people with 2+ employees |  | Goods and Services | NaNDA, Eating and Drinking Places by Census Tract, North American Industry Classification System and National Establishment Time Series, 2010 |
| Number of pharmacies per sq. mile with 2+ employees | Yes | Healthcare | NaNDA, Health Care Services by Census Tract, North American Industry Classification System and National Establishment Time Series, 2010 |
| Number of optical goods store per sq mile with 2+ employees | Yes | Healthcare | NaNDA, Health Care Services by Census Tract, North American Industry Classification System and National Establishment Time Series, 2010 |
| Number of misc. health/personal care stores per sq. mile with 2+ employees |  | Healthcare | NaNDA, Health Care Services by Census Tract, North American Industry Classification System and National Establishment Time Series, 2010 |
| Number of physicians (expect mental health) per sq. mile with 2+ employees | Yes | Healthcare | NaNDA, Health Care Services by Census Tract, North American Industry Classification System and National Establishment Time Series, 2010 |
| Number of mental health physicians per sq. mile with 2+ employees | Yes | Healthcare | NaNDA, Health Care Services by Census Tract, North American Industry Classification System and National Establishment Time Series, 2010 |
| Number of dentists per sq. mile with 2+ employees | Yes | Healthcare | NaNDA, Health Care Services by Census Tract, North American Industry Classification System and National Establishment Time Series, 2010 |
| Number of chiropractors per sq. mile with 2+ employees |  | Healthcare | NaNDA, Health Care Services by Census Tract, North American Industry Classification System and National Establishment Time Series, 2010 |
| Number of optometrists per sq. mile with 2+ employees |  | Healthcare | NaNDA, Health Care Services by Census Tract, North American Industry Classification System and National Establishment Time Series, 2010 |
| Number of mental health practitioners (non-physician) per sq. mile with 2+ employees |  | Healthcare | NaNDA, Health Care Services by Census Tract, North American Industry Classification System and National Establishment Time Series, 2010 |
| Number of physical, occupational, and speech therapists per sq. mile with 2+ employees |  | Healthcare | NaNDA, Health Care Services by Census Tract, North American Industry Classification System and National Establishment Time Series, 2010 |
| Number of other health practitioners per sq. mile with 2+ employees | Yes | Healthcare | NaNDA, Health Care Services by Census Tract, North American Industry Classification System and National Establishment Time Series, 2010 |
| Number of outpatient care centers per sq. mile with 2+ employees |  | Healthcare | NaNDA, Health Care Services by Census Tract, North American Industry Classification System and National Establishment Time Series, 2010 |
| Number of diagnostic labs per sq. mile with 2+ employees |  | Healthcare | NaNDA, Health Care Services by Census Tract, North American Industry Classification System and National Establishment Time Series, 2010 |
| Number of home health services per sq. mile with 2+ employees |  | Healthcare | NaNDA, Health Care Services by Census Tract, North American Industry Classification System and National Establishment Time Series, 2010 |
| Number of other ambulatory care services per sq. mile with 2+ employees | Yes | Healthcare | NaNDA, Health Care Services by Census Tract, North American Industry Classification System and National Establishment Time Series, 2010 |
| Number of nursing and residential care facilities per sq. mile with 2+ employees | Yes | Healthcare | NaNDA, Health Care Services by Census Tract, North American Industry Classification System and National Establishment Time Series, 2010 |
| Number of nursing care facilities per sq. mile with 2+ employees |  | Healthcare | NaNDA, Health Care Services by Census Tract, North American Industry Classification System and National Establishment Time Series, 2010 |
| Number of residential disability facilities per sq. mile with 2+ employees |  | Healthcare | NaNDA, Health Care Services by Census Tract, North American Industry Classification System and National Establishment Time Series, 2010 |
| Number of intellectual disability facilities per sq. mile with 2+ employees |  | Healthcare | NaNDA, Health Care Services by Census Tract, North American Industry Classification System and National Establishment Time Series, 2010 |
| Number of mental health and abuse facilities per sq. mile with 2+ employees |  | Healthcare | NaNDA, Health Care Services by Census Tract, North American Industry Classification System and National Establishment Time Series, 2010 |
| Number of continuing care and assisted living per sq. mile with 2+ employees |  | Healthcare | NaNDA, Health Care Services by Census Tract, North American Industry Classification System and National Establishment Time Series, 2010 |
| Number of other residential care facilities per sq. mile with 2+ employees |  | Healthcare | NaNDA, Health Care Services by Census Tract, North American Industry Classification System and National Establishment Time Series, 2010 |
| Number of pharmacies per 1000 people with 2+ employees |  | Healthcare | NaNDA, Health Care Services by Census Tract, North American Industry Classification System and National Establishment Time Series, 2010 |
| Number of optical goods store per 1000 people with 2+ employees |  | Healthcare | NaNDA, Health Care Services by Census Tract, North American Industry Classification System and National Establishment Time Series, 2010 |
| Number of misc. health/personal care stores per 1000 people with 2+ employees |  | Healthcare | NaNDA, Health Care Services by Census Tract, North American Industry Classification System and National Establishment Time Series, 2010 |
| Number of physicians (expect mental health) per 1000 people with 2+ employees | Yes | Healthcare | NaNDA, Health Care Services by Census Tract, North American Industry Classification System and National Establishment Time Series, 2010 |
| Number of mental health physicians per 1000 people with 2+ employees |  | Healthcare | NaNDA, Health Care Services by Census Tract, North American Industry Classification System and National Establishment Time Series, 2010 |
| Number of dentists per 1000 people with 2+ employees | Yes | Healthcare | NaNDA, Health Care Services by Census Tract, North American Industry Classification System and National Establishment Time Series, 2010 |
| Number of chiropractors per 1000 people with 2+ employees |  | Healthcare | NaNDA, Health Care Services by Census Tract, North American Industry Classification System and National Establishment Time Series, 2010 |
| Number of optometrists per 1000 people with 2+ employees |  | Healthcare | NaNDA, Health Care Services by Census Tract, North American Industry Classification System and National Establishment Time Series, 2010 |
| Number of mental health practitioners (non-physician) per 1000 people with 2+ employees |  | Healthcare | NaNDA, Health Care Services by Census Tract, North American Industry Classification System and National Establishment Time Series, 2010 |
| Number of physical, occupational, and speech therapists per 1000 people with 2+ employees |  | Healthcare | NaNDA, Health Care Services by Census Tract, North American Industry Classification System and National Establishment Time Series, 2010 |
| Number of other health practitioners per 1000 people with 2+ employees |  | Healthcare | NaNDA, Health Care Services by Census Tract, North American Industry Classification System and National Establishment Time Series, 2010 |
| Number of outpatient care centers per 1000 people with 2+ employees |  | Healthcare | NaNDA, Health Care Services by Census Tract, North American Industry Classification System and National Establishment Time Series, 2010 |
| Number of diagnostic labs per 1000 people with 2+ employees |  | Healthcare | NaNDA, Health Care Services by Census Tract, North American Industry Classification System and National Establishment Time Series, 2010 |
| Number of home health services per 1000 people with 2+ employees |  | Healthcare | NaNDA, Health Care Services by Census Tract, North American Industry Classification System and National Establishment Time Series, 2010 |
| Number of other ambulatory care services per 1000 people with 2+ employees |  | Healthcare | NaNDA, Health Care Services by Census Tract, North American Industry Classification System and National Establishment Time Series, 2010 |
| Number of nursing and residential care facilities per 1000 people with 2+ employees |  | Healthcare | NaNDA, Health Care Services by Census Tract, North American Industry Classification System and National Establishment Time Series, 2010 |
| Number of nursing care facilities per 1000 people with 2+ employees |  | Healthcare | NaNDA, Health Care Services by Census Tract, North American Industry Classification System and National Establishment Time Series, 2010 |
| Number of residential disability care facilities per 1000 people with 2+ employees |  | Healthcare | NaNDA, Health Care Services by Census Tract, North American Industry Classification System and National Establishment Time Series, 2010 |
| Number of intellectual disability care facilities per 1000 people with 2+ employees |  | Healthcare | NaNDA, Health Care Services by Census Tract, North American Industry Classification System and National Establishment Time Series, 2010 |
| Number of mental health and abuse care facilities per 1000 people with 2+ employees |  | Healthcare | NaNDA, Health Care Services by Census Tract, North American Industry Classification System and National Establishment Time Series, 2010 |
| Number of continuing care and assisted living per 1000 people with 2+ employees |  | Healthcare | NaNDA, Health Care Services by Census Tract, North American Industry Classification System and National Establishment Time Series, 2010 |
| Number of other residential care facilities per 1000 people with 2+ employees |  | Healthcare | NaNDA, Health Care Services by Census Tract, North American Industry Classification System and National Establishment Time Series, 2010 |
| One or more open parks in census tract |  | Recreation and Transportation | NaNDA, Parks by Census Tract, ParkServe, 2018 |
| Count of transit stops reported to National Transit Map as of 2018 | Yes | Recreation and Transportation | NaNDA, Public Transit Stops by Census Tract, National Transit Map, 2016 |
| Total number of open parks (top coded at 3) in census tract. 3 indicates 3 or more parks in the tract |  | Recreation and Transportation | NaNDA, Parks by Census Tract, ParkServe, 2018 |
| Density of performing arts companies in 2010 (count/pop) |  | Recreation and Transportation | NaNDA, Arts, Entertainment, and Recreation Organizations by Census Tract, North American Industry Classification System and National Establishment Time Series, 2010 |
| Density of spectator sports organizations in 2010 (counts/pop) |  | Recreation and Transportation | NaNDA, Arts, Entertainment, and Recreation Organizations by Census Tract, North American Industry Classification System and National Establishment Time Series, 2010 |
| Density of museums, historic sites, etc. in 2010 (counts/pop) |  | Recreation and Transportation | NaNDA, Arts, Entertainment, and Recreation Organizations by Census Tract, North American Industry Classification System and National Establishment Time Series, 2010 |
| Density of amusement parks/arcades in 2013 (count/pop) |  | Recreation and Transportation | NaNDA, Arts, Entertainment, and Recreation Organizations by Census Tract, North American Industry Classification System and National Establishment Time Series, 2010 |
| Density of gambling establishments in 2010 (counts/pop) |  | Recreation and Transportation | NaNDA, Arts, Entertainment, and Recreation Organizations by Census Tract, North American Industry Classification System and National Establishment Time Series, 2010 |
| Density of golf courses and country clubs in 2010 (counts/pop) |  | Recreation and Transportation | NaNDA, Arts, Entertainment, and Recreation Organizations by Census Tract, North American Industry Classification System and National Establishment Time Series, 2010 |
| Density of fitness/recreation centers in 2010 (counts/pop) |  | Recreation and Transportation | NaNDA, Arts, Entertainment, and Recreation Organizations by Census Tract, North American Industry Classification System and National Establishment Time Series, 2010 |
| Density of bowling alleys in 2010 (counts/pop) |  | Recreation and Transportation | NaNDA, Arts, Entertainment, and Recreation Organizations by Census Tract, North American Industry Classification System and National Establishment Time Series, 2010 |
| Density of casino hotels in 2010 (counts/pop) |  | Recreation and Transportation | NaNDA, Arts, Entertainment, and Recreation Organizations by Census Tract, North American Industry Classification System and National Establishment Time Series, 2010 |
| Proportion of open park land within census tract (total parks in area/area of the tract) |  | Recreation and Transportation | NaNDA, Parks by Census Tract, ParkServe, 2018 |
| Transit stops per 1000 people | Yes | Recreation and Transportation | NaNDA, Public Transit Stops by Census Tract, National Transit Map, 2016 |
| Transit stops per square mile | Yes | Recreation and Transportation | NaNDA, Public Transit Stops by Census Tract, National Transit Map, 2016 |
| Total area of open parks in the census tract, square miles |  | Recreation and Transportation | NaNDA, Parks by Census Tract, ParkServe, 2018 |
| Count of points (out of 24) more than 20 m uphill of the location |  | Urban Form | National Elevations Dataset |
| Count of points (out of 24) within 20 m of the same elevation |  | Urban Form | National Elevations Dataset |
| Count of points (out of 24) more than 20 m downhill of the location |  | Urban Form | National Elevations Dataset |
| Standard deviation of elevation of twenty points surrounding location | Yes | Urban Form | National Elevations Dataset |
| Elevation above sea level in meter | Yes | Urban Form | National Elevations Dataset |
| Sum of major emissions from tall stacks within 30 km, minus the emissions from tall stacks within 3 km |  | Urban Form | National Emissions Inventory 2008 |
| Sum of major emissions from tall stacks within 30 km, minus the emissions from tall stacks within 3 km |  | Urban Form | National Emissions Inventory 2008 |
| Impervious Surface - percentage of area in a 1km radius that is covered with an impervious surface such as pavement or concrete |  | Urban Form | Multi-Resolution Land Characteristics (MRLC) Consortium's National Landcover Dataset, 2006 |
| Number of A1/A1 intersections in 1000 m buffer |  | Urban Form | TeleAtlas 2000 |
| Number of A1/A2 intersections in 1000 m buffer |  | Urban Form | TeleAtlas 2000 |
| Number of A1/A3 intersections in 1000 m buffer |  | Urban Form | TeleAtlas 2000 |
| Number of A2/A2 intersections in 1000 m buffer |  | Urban Form | TeleAtlas 2000 |
| Number of A2/A3 intersections in 1000 m buffer |  | Urban Form | TeleAtlas 2000 |
| Number of A3/A3 intersections in 1000 m buffer | Yes | Urban Form | TeleAtlas 2000 |
| Length of A2 roads within 1000 m buffer |  | Urban Form | TeleAtlas 2000 |
| Length of A3 roads within 1000 m buffer |  | Urban Form | TeleAtlas 2000 |
| Meters to where A1 roads intersect | Yes | Urban Form | TeleAtlas 2000 |
| Meters to where A1 and A3 roads intersect | Yes | Urban Form | TeleAtlas 2000 |
| Meters to where A2 and A3 roads intersect | Yes | Urban Form | TeleAtlas 2000 |
| Meters to A3 road | Yes | Urban Form | TeleAtlas 2000 |
| Meters to where A3 and A3 roads intersect | Yes | Urban Form | TeleAtlas 2000 |
| Meters to Airport |  | Urban Form | NEI Database |
| Meters to Coastline | Yes | Urban Form | TeleAtlas 2000 |
| Meters to Large Airport |  | Urban Form | NEI Database |
| Meters to a large port |  | Urban Form | National Geospatial Intelligence Agency |
| Meters to a medium port |  | Urban Form | National Geospatial Intelligence Agency |
| Meters to Railroads | Yes | Urban Form | TeleAtlas 2000 |
| Meters to Railyards | Yes | Urban Form | TeleAtlas 2000 |
| Meters to small port |  | Urban Form | National Geospatial Intelligence Agency |
| Meters to truck route | Yes | Urban Form | National Transportation Atlas Database 2009 |
| NDVI Summer in 1km buffer | Yes | Urban Form | University of Maryland, 2006 |
| NDVI Winter in 1km buffer | Yes | Urban Form | University of Maryland, 2006 |
| Bare rock, sand, barren, mine in 1km radius raster |  | Urban Form | Multi-Resolution Land Characteristics (MRLC) Consortium's National Landcover Dataset, 2006 |
| Cultivated crops such as orchards, vineyards, grains in 1km radius raster |  | Urban Form | Multi-Resolution Land Characteristics (MRLC) Consortium's National Landcover Dataset, 2006 |
| Deciduous forest in 1km radius raster |  | Urban Form | Multi-Resolution Land Characteristics (MRLC) Consortium's National Landcover Dataset, 2006 |
| Developed high intensity in 1km radius raster |  | Urban Form | Multi-Resolution Land Characteristics (MRLC) Consortium's National Landcover Dataset, 2006 |
| Developed low intensity in 1km radius raster | Yes | Urban Form | Multi-Resolution Land Characteristics (MRLC) Consortium's National Landcover Dataset, 2006 |
| Developed medium intensity in 1km radius raster |  | Urban Form | Multi-Resolution Land Characteristics (MRLC) Consortium's National Landcover Dataset, 2006 |
| Developed open space in 1km radius raster | Yes | Urban Form | Multi-Resolution Land Characteristics (MRLC) Consortium's National Landcover Dataset, 2006 |
| Evergreen forest in 1km radius raster |  | Urban Form | Multi-Resolution Land Characteristics (MRLC) Consortium's National Landcover Dataset, 2006 |
| Grasslands, herbaceous vegetation in 1km radius raster |  | Urban Form | Multi-Resolution Land Characteristics (MRLC) Consortium's National Landcover Dataset, 2006 |
| Emergent herbaceous wetland in 1km radius raster |  | Urban Form | Multi-Resolution Land Characteristics (MRLC) Consortium's National Landcover Dataset, 2006 |
| Mixed forest in 1km radius raster |  | Urban Form | Multi-Resolution Land Characteristics (MRLC) Consortium's National Landcover Dataset, 2006 |
| Pasture, hay in 1km radius raster |  | Urban Form | Multi-Resolution Land Characteristics (MRLC) Consortium's National Landcover Dataset, 2006 |
| Shrubland in 1km radius raster |  | Urban Form | Multi-Resolution Land Characteristics (MRLC) Consortium's National Landcover Dataset, 2006 |
| Open water in 1km radius raster |  | Urban Form | Multi-Resolution Land Characteristics (MRLC) Consortium's National Landcover Dataset, 2006 |
| Woody wetlands in 1km radius raster |  | Urban Form | Multi-Resolution Land Characteristics (MRLC) Consortium's National Landcover Dataset, 2006 |
| Length of Truck Route within 1 km buffer |  | Urban Form | National Transportation Atlas Database 2009 |
| Tract area in sq. miles |  | Urban Form | 2010 US Census |
| Note: NaNDA – National Neighborhood Data Archive | | | |
