# Supplementary material for: Neighborhood environment and incident diabetes, a neighborhood environment-wide association study (‘NE-WAS’): Results from the Hispanic Community Health Study/Study of Latinos (HCHS/SOL)
Source: PLoS One. 2025 Jul 29;20(7):e0329282. doi: 10.1371/journal.pone.0329282 (PMC12306752; doi:10.1371/journal.pone.0329282)
Supplement: S2 Table — (DOCX) [file pone.0329282.s002.docx]

**S2 Table.** Characteristics of participants included in analysis of primary diabetes outcome (N=8006) by study center.

| **Variables** |  | Sample Weighted % or Mean (SD) | |  |
| --- | --- | --- | --- | --- |
|  | The Bronx (n=1561) | Chicago  (n= 2255) | Miami  (n=1927) | San Diego  (n=2263) |
| **Female** | 53.2 | 47.1 | 48.9 | 51.6 |
| **Age, years** | 38.4 (13.8) | 36.8 (12.7) | 43.0 (14.0) | 37.5 (13.8) |
| **Waist Circumference (cm)** | 96.2 (14.5) | 96.0 (13.6) | 95.4 (12.8) | 97.5 (13.8) |
| **Hispanic/Latino Heritage** |  |  |  |  |
| Dominican | 30.9 | 0.5 | 1.5 | 0.1 |
| Central American | 4.8 | 6.8 | 15.7 | 1.0 |
| Cuban | 1.0 | 1.1 | 65.7 | 0.4 |
| Mexican | 12.9 | 61.6 | 1.4 | 92.1 |
| Puerto Rican | 38.9 | 20.0 | 2.3 | 1.8 |
| South American | 4.4 | 7.1 | 9.1 | 0.9 |
| More than one heritage | 5.8 | 2.8 | 3.8 | 2.5 |
| Other | 1.1 | 0.1 | 0.4 | 1.3 |
| **Years in the US** |  |  |  |  |
| Less than 10 years | 16.7 | 23.4 | 46.9 | 21.6 |
| 10 years or more | 51.1 | 54.0 | 44.4 | 44.5 |
| US Born | 32.2 | 22.6 | 8.7 | 33.9 |
| **Family History of Diabetes** | 40.9 | 41.5 | 30.4 | 39.2 |
| **Marital Status** |  |  |  |  |
| Single | 48.6 | 31.7 | 29.6 | 30.7 |
| Married or living with a Partner | 36.7 | 57.3 | 49.8 | 58.0 |
| Separated, Divorced, or Widow | 14.7 | 11.1 | 20.6 | 11.3 |
| **Education** |  |  |  |  |
| No High School Diploma or GED | 33.9 | 38.4 | 19.2 | 25.8 |
| At most a High school diploma or GED | 28.7 | 32.6 | 28.4 | 28.3 |
| High school (or GED) education | 5.0 | 8.2 | 20.7 | 16.7 |
| University/college education | 32.4 | 20.8 | 31.7 | 29.2 |
| **Income** |  |  |  |  |
| Less than $10,000 | 18.8 | 7.8 | 17.7 | 8.9 |
| $10,001-$20,000 | 34.9 | 31.0 | 36.5 | 23.7 |
| $20,001-$40,000 | 29.2 | 40.5 | 31.6 | 35.8 |
| $40,001-$75,000 | 13.6 | 15.0 | 10.5 | 21.5 |
| More than $75,000 | 3.5 | 5.6 | 3.6 | 10.1 |
| **Years between Visit 1 and Visit 2** | 6.1 (0.93) | 6.2 (0.78) | 5.9 (0.88) | 6.3 (0.79) |
| **Visit 2 Diabetes Status** |  |  |  |  |
| Diabetes (blood test and self-report medication | 10.3 | 9.6 | 8.2 | 8.7 |
| Diabetes (blood test, self-report medication, and self-reported diagnosis) | 13.7 | 13.8 | 10.6 | 15.9 |
