# Supplementary figures and images for: Neighborhood environment and incident diabetes, a neighborhood environment-wide association study (‘NE-WAS’): Results from the Hispanic Community Health Study/Study of Latinos (HCHS/SOL)
Source: PLoS One. 2025 Jul 29;20(7):e0329282. doi: 10.1371/journal.pone.0329282 (PMC12306752; doi:10.1371/journal.pone.0329282)

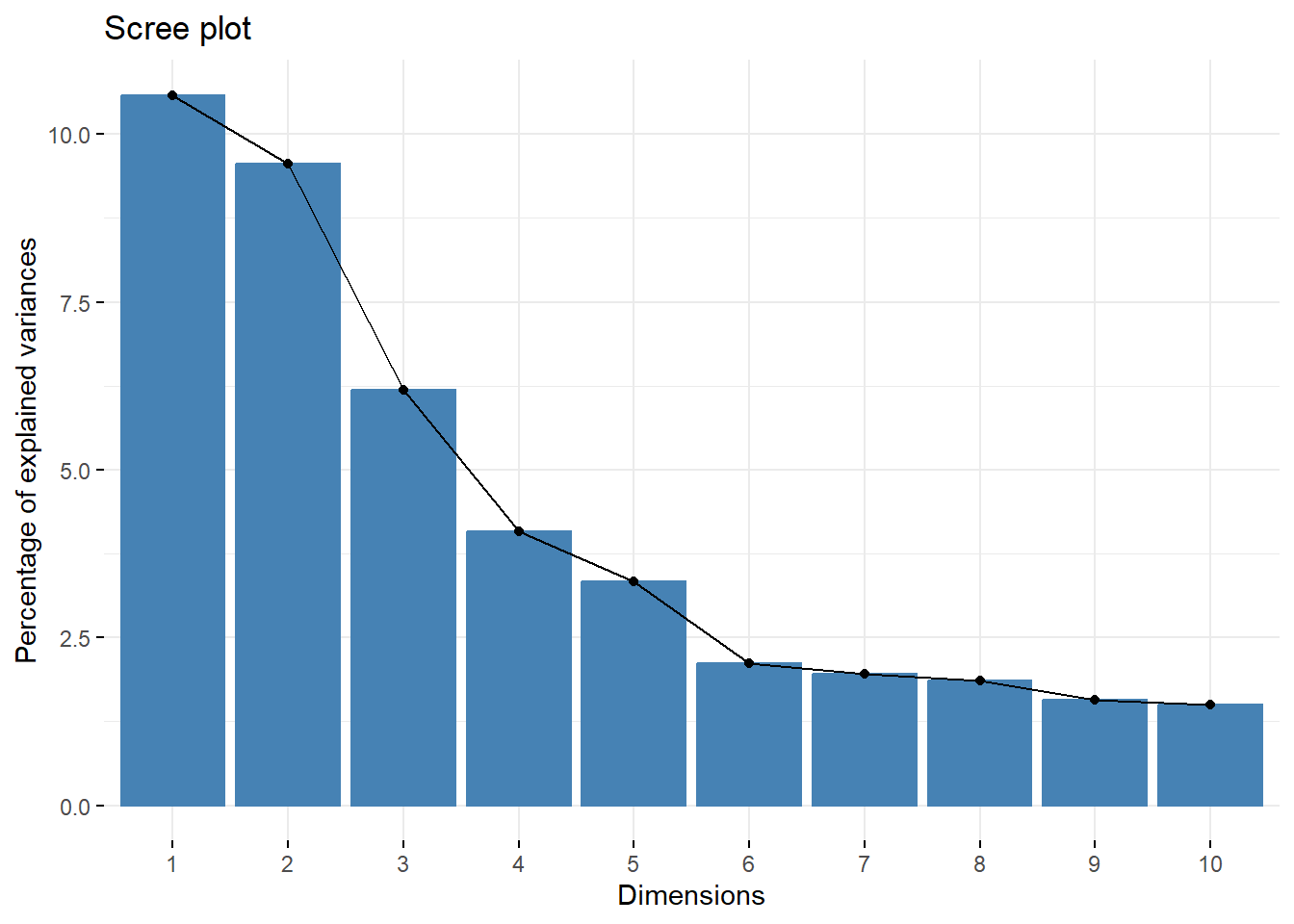

Supplement: S3 Fig — (TIF) [file pone.0329282.s003.tif]
