# Supplementary material for: Neighborhood environment and incident diabetes, a neighborhood environment-wide association study (‘NE-WAS’): Results from the Hispanic Community Health Study/Study of Latinos (HCHS/SOL)
Source: PLoS One. 2025 Jul 29;20(7):e0329282. doi: 10.1371/journal.pone.0329282 (PMC12306752; doi:10.1371/journal.pone.0329282)
Supplement: S4 Table — (DOCX) [file pone.0329282.s004.docx]

**S4 Table.** Incidence rate ratio (IRR) from Poisson regression of the first two principal components and incident diabetes adjusting for age, sex, education, income, marital status, ethnic background, years in US, family history of diabetes and accounting for survey weights and design.

| **Variable** | **IRR** | **95% CI** |
| --- | --- | --- |
|  |  |  |
| **PC1** | 1.01 | 0.99 – 1.03 |
| **PC2** | 1.01 | 0.99 – 1.03 |
| **Age** | 1.04 | 1.03 – 1.05 * |
| **Sex_Male** | 1.04 | 1.03 – 1.04 |
| **Education_ No high school diploma (reference)** |  |  |
| **Education_ At most high school diploma or GED** | 0.93 | 0.72 – 1.20 |
| **Education_ High School or GED** | 0.77 | 0.56 – 1.05 |
| **Education_ University/college education** | 0.73 | 0.53 – 1.00* |
| **Income_ Less than $10,000 (reference)** |  |  |
| **Income_ $10,001-$20,00** | 1.08 | 0.82 – 1.42 |
| **Income_ $20,001 - $40,000** | 1.09 | 0.83 – 1.42 |
| **Income_ $40,001 - $75,00** | 0.94 | 0.67 – 1.33 |
| **Income_ More than $75,000** | 1.17 | 0.74 – 1.86 |
| **Martial_ Single (reference)** |  |  |
| **Martial_ Married or living with a partner** | 1.26 | 0.94 – 1.67 |
| **Martial_ Separated, divorced, or widow(er)** | 1.14 | 0.83 – 1.56 |
| **Heritage – Dominican (reference)** |  |  |
| **Heritage – Central American** | 0.67 | 0.43 – 1.03 |
| **Heritage - Cuban** | 0.85 | 0.58 – 1.27 |
| **Heritage - Mexican** | 0.93 | 0.65 – 1.33 |
| **Heritage – Puerto Rican** | 1.01 | 0.67 – 1.53 |
| **Heritage – South American** | 0.64 | 0.38 – 1.08 |
| **Heritage – More than one heritage** | 1.82 | 1.02 – 3.27* |
| **Heritage - Other** | 0.26 | 0.08 – 0.87* |
| **Years in US – US born (reference)** |  |  |
| **Less than 10 years** | 0.97 | 0.66 – 1.43 |
| **10 or more years** | 0.97 | 0.72 – 1.31 |
| **Family History of Diabetes** | 1.50 | 1.24 – 1.82* |

Note: * p-value < 0.05. IRR = Incidence Rate Ratio
