# Supplementary material for: Neighborhood environment and incident diabetes, a neighborhood environment-wide association study (‘NE-WAS’): Results from the Hispanic Community Health Study/Study of Latinos (HCHS/SOL)
Source: PLoS One. 2025 Jul 29;20(7):e0329282. doi: 10.1371/journal.pone.0329282 (PMC12306752; doi:10.1371/journal.pone.0329282)
Supplement: S5 Table — (DOCX) [file pone.0329282.s005.docx]

**S5 Table.** Characteristics of participants who did not move during follow up (N= 4188) and those who did move (N= 3818).

|  | Sample Weighted % or Mean (SD) | |  |
| --- | --- | --- | --- |
| **Variables** | Do Not Move (N= 4188) | Moved (N=3818) | Test Statistic |
| **Female** | 51.7 | 49.4 | X^2^ = 2.10 |
| **Age, years** | 41.6 (14.3) | 37.0 (13.1) | T = 9.35* |
| **Waist Circumference (cm)** | 96.8 (13.5) | 95.9 (13.9) | T = 1.81 |
| **Hispanic/Latino Heritage** |  |  |  |
| Central American | 10.5 | 6.52 | X^2^= 7.0* |
| Cuban | 6.49 | 7.75 |  |
| Dominican | 15.0 | 23.0 |  |
| Mexican | 41.6 | 40.3 |  |
| Puerto Rican | 16.9 | 12.4 |  |
| South American | 5.10 | 5.28 |  |
| More than one heritage | 3.41 | 4.11 |  |
| Other | 0.97 | 0.63 |  |
| **Years in the US** |  |  |  |
| Less than 10 years | 20.9 | 34.1 | X^2^ = 35.0* |
| 10 years or more | 55.0 | 41.3 |  |
| US Born | 24.2 | 24.6 |  |
| **Family History of Diabetes** | 39.4 | 35.8 | X^2^ = 4.81* |
| **Marital Status** |  |  |  |
| Single | 33.2 | 36.8 | X^2^ = 2.43 |
| Married or living with a Partner | 52.5 | 48.9 |  |
| Separated, Divorced, or Widow | 15.2 | 14.3 |  |
| **Education** |  |  |  |
| No High School Diploma or GED | 28.7 | 27.8 | X^2^ = 1.96 |
| At most a High school diploma or GED | 27.8 | 30.4 |  |
| High school (or GED) education | 12.7 | 14.0 |  |
| University/college education | 30.8 | 27.9 |  |
| **Income** |  |  |  |
| Less than $10,000 | 13.2 | 14.1 | X^2^= 4.52* |
| $10,001-$20,000 | 29.0 | 33.7 |  |
| $20,001-$40,000 | 33.9 | 33.6 |  |
| $40,001-$75,000 | 16.7 | 14.0 |  |
| More than $75,000 | 7.15 | 4.58 |  |
| **Years between Visit 1 and Visit 2** | 6.07 (0.83) | 6.19 (0.89) | T = -3.26* |
| **Study Center** |  |  |  |
| Bronx | 32.4 | 19.1 | X^2^ = 28.6* |
| Chicago | 14.7 | 20.1 |  |
| Miami | 22.0 | 34.1 |  |
| San Diego | 30.9 | 26.8 |  |
| **Visit 2 Diabetes Status** |  |  |  |
| Diabetes (blood test and self-report medication | 10.4 | 8.03 | X^2^=6.79* |
