# Supplementary material for: Neighborhood environment and incident diabetes, a neighborhood environment-wide association study (‘NE-WAS’): Results from the Hispanic Community Health Study/Study of Latinos (HCHS/SOL)
Source: PLoS One. 2025 Jul 29;20(7):e0329282. doi: 10.1371/journal.pone.0329282 (PMC12306752; doi:10.1371/journal.pone.0329282)
Supplement: S6 Table — (DOCX) [file pone.0329282.s006.docx]

**S6 Table.** Characteristics of participants with incident diabetes based on the primary (N=926) and secondary definition (N=1323).

|  | Sample Weighted % or Mean (SD) | |  |
| --- | --- | --- | --- |
| **Variables** | Diabetes 4 | Diabetes 5 | Test Statistic |
| **Incident Cases** | 926 | 1323 |  |
| **Female** | 46.9 | 50.5 | X^2^ = 5.70* |
| **Age, years** | 45.9 (12.6) | 44.9 (12.9) | T = 1.98* |
| **Waist Circumference (cm)** | 105.3 (13.4) | 103.2 (13.9) | T = 4.85* |
| **Hispanic/Latino Heritage** |  |  |  |
| Central American | 8.80 | 6.63 | X^2^ = 4.57* |
| Cuban | 4.74 | 4.08 |  |
| Dominican | 20.0 | 17.3 |  |
| Mexican | 40.0 | 43.8 |  |
| Puerto Rican | 18.0 | 19.9 |  |
| South American | 3.37 | 3.03 |  |
| More than one heritage | 4.92 | 4.07 |  |
| Other | 0.24 | 1.14 |  |
| **Years in the US** |  |  |  |
| Less than 10 years | 23.9 | 23.2 | X^2^ = 1.58 |
| 10 years or more | 57.0 | 55.2 |  |
| US Born | 19.1 | 21.6 |  |
| **Family History of Diabetes** | 49.5 | 49.2 | X^2^ = 0.02 |
| **Marital Status** |  |  |  |
| Single | 23.5 | 23.7 | X^2^ = 0.45 |
| Married or living with a Partner | 57.3 | 58.3 |  |
| Separated, Divorced, or Widow | 19.1 | 17.9 |  |
| **Education** |  |  |  |
| No High School Diploma or GED | 36.2 | 34.3 | X^2^ = 1.72 |
| At most a High school diploma or GED | 27.9 | 27.6 |  |
| High school (or GED) education | 11.9 | 11.2 |  |
| University/college education | 24.0 | 26.9 |  |
| **Income** |  |  |  |
| Less than $10,000 | 14.7 | 15.4 | X^2^ = 0.53 |
| $10,001-$20,000 | 31.9 | 32.5 |  |
| $20,001-$40,000 | 34.0 | 31.8 |  |
| $40,001-$75,000 | 13.3 | 14.0 |  |
| More than $75,000 | 6.15 | 6.34 |  |
| **Years between Visit 1 and Visit 2** | 6.07 (0.83) | 6.17 (0.92) | T = -2.58* |
| **Study Center** |  |  |  |
| Bronx | 28.7 | 26.1 | X^2^ = 6.15* |
| Chicago | 18.3 | 17.7 |  |
| Miami | 25.6 | 22.3 |  |
| San Diego | 27.4 | 33.8 |  |
